# Rs-fMRI reveals that taVNS improves cognitive and emotional function in mild cognitive impairment by reconfiguration of “sensory relay-salience-default” cross-network effective connectivity: a randomized, double-blind, sham-controlled trial

**DOI:** 10.3389/fneur.2026.1891440

**Published:** 2026-08-12

**Authors:** Tianjiao Xu, Chunlei Guo, Lei Zhang, Jiudong Cao, Mingyv Li, Jiazheng Li, Kuncheng Li, Jin Yang, Jiliang Fang

**Affiliations:** 1 Department of Radiology, Guang’anmen Hospital, China Academy of Chinese Medical Sciences, Beijing, China; 2 Department of Acupuncture, Guang’anmen Hospital, China Academy of Chinese Medical Sciences, Beijing, China; 3 Department of Asset Management Section Medical Engineering, Guang’anmen Hospital, China Academy of Chinese Medical Sciences, Beijing, China

**Keywords:** brain networks, functional connectivity, functional magnetic resonance imaging, granger causality analysis, mild cognitive impairment, transcutaneous auricular vagus nerve stimulation

## Abstract

**Background:**

Transcutaneous auricular vagus nerve stimulation (taVNS) is a promising noninvasive intervention for mild cognitive impairment (MCI), but its clinical efficacy and network-level mechanisms remain unclear.

**Methods:**

In this prospective, randomized, sham-controlled, parallel-group trial, 60 patients with MCI were assigned 1:1 to 12-week taVNS or sham taVNS. The primary outcome was the between-group difference in change from baseline to week 12 on the Montreal Cognitive Assessment-Basic (MoCA-B). Secondary outcomes included AVLT-H, STT-A/B, AFT, BNT, HAMD-17, and HAMA scores. Twenty-eight healthy controls underwent baseline rs-fMRI as a non-randomized reference cohort. Functional connectivity (FC) identified MCI-related abnormal regions, which together with theory-driven regions served as seeds for Granger causality analysis (GCA) to characterize intervention-related effective connectivity changes. Imaging-behavior associations were examined using Pearson correlations. The final per-protocol analysis included 52 randomized patients with complete clinical and imaging data.

**Results:**

After 12 weeks, the taVNS group showed significant within-group improvements in global cognition and multiple secondary outcomes, whereas the sham group did not. The between-group difference in MoCA-B change was significant (mean difference = 2.91, 95% CI: 0.79 to 5.03, Cohen’s d = 0.59), indicating a moderate-to-large effect. Baseline FC abnormalities in MCI mainly involved the insula, medial frontal cortex, precuneus, temporal cortex, thalamus, cingulate cortex, and parieto-occipital regions. Following taVNS, effective connectivity was selectively reconfigured within a distributed network centered on the insula, anterior cingulate cortex, temporal cortex, and precuneus. Changes in directed connectivity correlated with changes in MoCA-B, AFT, and HAMA scores. No serious adverse events occurred.

**Clinical trial registration:**

https://www.chictr.org.cn/showproj.html?proj=131823, identifier (ChiCTR2100049940).

## 1 Introduction

Mild cognitive impairment (MCI) is defined by objective cognitive decline with preserved functional independence and is widely considered a prodromal stage with increased risk of progression to dementia (1–3). This elevated conversion risk underscores the need for early interventions grounded in plausible neurobiological mechanisms. Accumulating evidence indicates that MCI involves large-scale dysregulation of distributed brain networks rather than focal abnormalities alone (4, 5). The default mode network (DMN) is most consistently affected, together with disruptions of the salience network (SN) and frontoparietal/executive control systems (6, 7). Key regions within these networks—including the precuneus/posterior cingulate cortex, medial prefrontal cortex, temporal cortex, insula, and cingulate cortex—are critical for memory, semantic processing, and emotional integration (8, 9). Elucidating abnormal cross-network information transfer and its treatment-related plasticity may therefore deepen understanding of MCI pathophysiology and inform early intervention development (10).

Transcutaneous auricular vagus nerve stimulation (taVNS) is a noninvasive, safe, and practical neuromodulatory approach that has gained increasing interest as a potential treatment for cognitive and affective disorders (11, 12). Previous studies suggest taVNS may improve attention, memory, and emotional symptoms while modulating neural activity in regions involved in memory, emotion regulation, and large-scale functional networks (13, 14). Neuroanatomically, stimulation of the auricular branch of the vagus nerve activates ascending pathways from the nucleus tractus solitarius (NTS) to multiple cortical and subcortical areas, including the thalamus, insula, anterior cingulate cortex, and prefrontal cortex (15, 16). These regions support salience detection, interoceptive integration, cognitive control, and emotional processing (16). Importantly, this ascending vagal pathway substantially overlaps with brain systems implicated in MCI, raising the possibility that taVNS exerts therapeutic effects by reshaping cross-network effective connectivity and modulating directed information flow across large-scale brain networks (17, 18).

Despite its promise, the central mechanisms by which taVNS improves cognitive and emotional dysfunction in MCI remain poorly understood (19). Most previous neuroimaging studies have focused on resting-state functional connectivity (20, 21), whereas much less is known about how taVNS influences directed interactions among brain regions—including the balance of driving and receiving nodes within and across networks—and how such changes relate to clinical improvement (22, 23). To address this gap, the present study combined functional connectivity (FC) analysis with Granger causality analysis (GCA) to systematically examine the effects of a 12-week taVNS intervention on abnormal resting-state brain networks in MCI. We also explored associations between treatment-related changes in effective connectivity and improvements in cognitive and emotional outcomes. We hypothesized that taVNS would be associated with amelioration of cognitive and emotional dysfunction in MCI and with reorganization of cross-network effective connectivity centered on insula-, anterior cingulate-, and precuneus-related pathways, with baseline involvement of relay- and salience-related regions corresponding to a putative “sensory relay–salience–default” axis.

## 2 Methods

### 2.1 Study design and participants

This was a prospective, double-blind, randomized, sham-controlled, parallel-group clinical trial. Between January 2021 and December 2023, patients with mild cognitive impairment (MCI) were recruited from Xuanwu Hospital, Capital Medical University, and Guang’anmen Hospital, China Academy of Chinese Medical Sciences. Eligible patients were randomly assigned in a 1:1 ratio to either the transcutaneous auricular vagus nerve stimulation (taVNS) group or the sham taVNS group. In addition, age-, sex-, and education-matched healthy controls (HCs) were recruited from the community as a non-randomized observational reference cohort for baseline imaging comparisons only. The study was conducted in accordance with the Declaration of Helsinki and was approved by the Ethics Committee of Guang’anmen Hospital, China Academy of Chinese Medical Sciences (Approval No. [2021-077-KY-01]). The trial was registered in the Chinese Clinical Trial Registry (ChiCTR2100049940). All participants provided written informed consent before enrollment. No important methodological changes were made after trial commencement.

### 2.2 Eligibility criteria

#### 2.2.1 Inclusion criteria

Participants were eligible if they met all of the following criteria: (1) age 55–80 years; (2) right-handed; (3) Han Chinese and local permanent residents who had lived in the area for at least 6 months after enrollment; (4) no evidence of cerebrovascular disease, such as ischemic stroke or intracranial aneurysm, on neuroimaging; (5) cognitive impairment meeting either the Jak/Bondi criteria, defined as scores at least 1.0 standard deviation (SD) below the age-adjusted Chinese normative mean on at least two neuropsychological test measures within any one cognitive domain (memory, language, or executive function), or the International Working Group criteria, defined as scores at least 1.0 SD below the age-adjusted Chinese normative mean on at least one test measure in each of the three cognitive domains of memory, language, and executive function; (6) willingness to participate and provision of written informed consent; and (7) no use of analgesics, anesthetics, or hypnotics within 1 month before screening.

#### 2.2.2 Exclusion criteria

Exclusion criteria were: (1) neurological disorders that may cause cognitive decline, such as encephalitis, brain tumor, traumatic brain injury, epilepsy, or Parkinson’s disease; (2) metabolic disorders associated with cognitive impairment, such as anemia, thyroid dysfunction, folate deficiency, or vitamin B12 deficiency; (3) severe psychiatric disorders, such as major depressive disorder or bipolar disorder; (4) history of carbon monoxide poisoning; (5) history of general anesthesia; (6) diagnosed dementia; (7) acute or life-threatening severe illness at enrollment; (8) severe visual, hearing, or language impairment precluding neuropsychological assessment; (9) contraindications to MRI, including a cardiac pacemaker, metallic implants, or claustrophobia; and (10) participation in another clinical study within the previous 6 months.

### 2.3 Clinical assessments

All participants underwent comprehensive neuropsychological and clinical assessments at baseline, and patients with MCI were reassessed after the 12-week intervention period. Cognitive function was evaluated using the Montreal Cognitive Assessment-Basic (MoCA-B), Auditory Verbal Learning Test-Huashan version (AVLT-H), Shape Trail Test Part A and Part B (STT-A and STT-B), Animal Fluency Test (AFT), and Boston Naming Test (BNT). Emotional status was assessed using the 17-item Hamilton Depression Rating Scale (HAMD-17) and the Hamilton Anxiety Rating Scale (HAMA). Participants were also asked to record treatment duration, stimulation intensity, adverse reactions, and any concomitant medication use throughout the intervention period. All assessments were conducted using standardized procedures.

### 2.4 Outcomes

The primary outcome was the between-group difference in change in Montreal Cognitive Assessment-Basic (MoCA-B) score from baseline to week 12. Secondary clinical outcomes included changes in AVLT-H, STT-A, STT-B, AFT, BNT, HAMD-17, and HAMA scores over the same period. Exploratory imaging outcomes included: (1) baseline whole-brain functional connectivity (FC) differences between patients with MCI and healthy controls; (2) pre- to post-intervention changes in Granger causality analysis (GCA)-based effective connectivity within the randomized MCI groups; and (3) correlations between changes in imaging metrics and changes in clinical scale scores. All clinical outcomes were assessed at baseline and week 12. No changes were made to the prespecified outcomes after trial commencement.

### 2.5 Randomization, allocation concealment, and blinding

This study used a randomized, double-blind, parallel-controlled design. Eligible patients were randomly assigned in a 1:1 ratio to the taVNS group or sham taVNS group using a computer-generated randomization schedule. The allocation sequence was generated and retained by personnel who were not involved in participant recruitment, intervention delivery, outcome assessment, or statistical analysis, thereby ensuring allocation concealment. Participants and statisticians were blinded to group assignment. Clinical assessments were conducted using standardized procedures, and group allocation was not disclosed during data analysis. The healthy control group was not randomized and was included only for baseline imaging comparison.

### 2.6 Intervention

In the taVNS group, stimulation was delivered to the bilateral cymba conchae regions corresponding to the heart and kidney areas, where the auricular branch of the vagus nerve is distributed. In the sham taVNS group, stimulation was delivered to the bilateral superior scapha and the middle segment of the descending helix, which are considered non-vagal auricular regions. Both groups received stimulation using the same device (Huatuo TENS-200A) with identical parameters: dense wave, 20 Hz; pulse width < 1 ms; intensity 4–6 mA adjusted to individual tolerance; 30 min per session, twice daily, 5 days per week, for 12 consecutive weeks. From the day of enrollment, all participants were required to record stimulation duration, stimulation intensity, adverse events, and concomitant medication use on a daily basis.

### 2.7 Sample size

This study was designed as an exploratory mechanistic randomized controlled trial. The target sample size was determined with reference to previous taVNS-related clinical and rs-fMRI studies in MCI (24, 25), together with practical feasibility during the recruitment period, rather than by a formal *a priori* power calculation—which is consistent with the exploratory nature of this investigation and the lack of reliable effect size estimates in the taVNS-fMRI literature at the time of trial design.

To evaluate whether the current sample provided sufficient statistical power to detect meaningful effects despite the absence of a formal pre-study power calculation, we performed post-hoc sensitivity analyses. Using G*Power software with the prespecified statistical criteria (two-tailed test, *α* = 0.05, power = 0.80) and the actual sample sizes (taVNS group: *n* = 29; sham group: *n* = 23), the minimum detectable effect size for the primary between-group comparison was Cohen’s d = 0.78, which falls within the range of a large effect size. For the within-group correlation analyses in the taVNS group (*n* = 29), the minimum detectable correlation coefficient was *r* = 0.367. All significant correlations reported in this study (r values ranging from −0.591 to 0.500, absolute values all > 0.367) exceeded this threshold, indicating adequate power to detect the observed medium-to-large effects.

Accordingly, the trial should be regarded as hypothesis-generating, particularly for the imaging and correlation analyses. The post-hoc sensitivity results are further reported in the Results section (Section 3.1).

### 2.8 MRI data acquisition

MRI data were acquired at the Department of Radiology, Guang’anmen Hospital, China Academy of Chinese Medical Sciences, using a 3.0-T Siemens Skyra scanner with a 20-channel head coil. Patients with MCI underwent MRI scanning at baseline and after 12 weeks of treatment, whereas healthy controls underwent a single baseline MRI scan. Total scan time was approximately 30 min per session.

Before scanning, participants removed all metallic objects and wore disposable earplugs. During scanning, the head was stabilized with foam padding, and participants were instructed to lie still with their eyes closed and remain awake.

The MRI sequences and acquisition parameters were as follows: (1) 3D T1-weighted structural imaging: FSPGR sequence, TR/TE = 15/6.9 ms, flip angle = 15°, matrix = 256 × 256, field of view = 200 mm, slice thickness = 1.4 mm, acquisition time = 8 min; (2) resting-state BOLD-fMRI: echo-planar imaging sequence, TR/TE = 2000/30 ms, flip angle = 90°, matrix = 64 × 64, field of view = 240 mm, slice thickness = 3.0 mm, interslice gap = 0.5 mm, number of slices = 41, voxel size = 3.5 × 3.5 × 3.0 mm^3^. The scan orientation was parallel to the anterior commissure-posterior commissure line, covered the whole brain, and included 4 dummy scans. Resting-state scan time was 6 min, which is within the range commonly used in prior rs-fMRI studies of MCI and neuromodulation (26–28). Previous methodological work has shown that 5–6 min of resting-state data can yield stable estimates of functional connectivity at the group level, although longer acquisitions may improve individual-level reliability.

### 2.9 Imaging preprocessing and analysis

MRI data were preprocessed using SPM12^1^ and DPABI (Version 8.1)^2^ (26). The preprocessing pipeline included the following steps (27, 28): Removal of initial volumes: The first 10 time points of each functional run were discarded to allow for signal equilibrium and participant adaptation to the scanning environment. Slice-timing correction: The remaining functional images were corrected for inter-slice acquisition time differences using the slice-timing correction procedure in SPM12, with sinc temporal interpolation to resample each slice’s BOLD time series to a common mid-acquisition time point. Head-motion correction: All functional images were realigned to the first volume using a rigid-body transformation (6 degrees of freedom) to correct for head motion. Participants with head motion exceeding 2.0 mm in translation or 2.0° in rotation were excluded from further analysis. The mean and standard deviation of head motion parameters (translation and rotation) for the included participants, as well as the mean framewise displacement (FD), are reported in the Supplementary Table S3 to provide quantitative quality-control metrics. Spatial normalization: The mean functional image was co-registered to each participant’s T1-weighted structural image. The structural images were segmented and spatially normalized to the standard Montreal Neurological Institute (MNI) echo-planar imaging template using the DARTEL (Diffeomorphic Anatomical Registration Through Exponentiated Lie Algebra) algorithm implemented in SPM12. The resulting deformation parameters were then applied to all functional images, resampling them to a voxel size of 3 × 3 × 3 mm^3^. Spatial smoothing: The normalized functional images were spatially smoothed using a 3D isotropic Gaussian kernel with a full width at half maximum (FWHM) of 6 mm to improve the signal-to-noise ratio and increase the validity of statistical inferences. Linear trend removal: A linear drift was removed from the time series of each voxel to eliminate low-frequency signal drift unrelated to neural activity. Nuisance covariate regression: Nuisance signals were regressed out from the functional data, including: (a) head-motion parameters using the Friston 24-parameter model (6 rigid-body parameters plus their temporal derivatives and squared terms); (b) signals from white matter and cerebrospinal fluid masks; and (c) global brain signal, to reduce the influence of physiological noise and non-neuronal fluctuations. Bandpass filtering: Finally, the residual time series were bandpass filtered within a frequency range of 0.01–0.08 Hz to minimize the effects of low-frequency drift and high-frequency physiological noise (e.g., cardiac and respiratory signals), retaining signals primarily of neuronal origin.

Based on previous studies (24, 29, 30), automated Anatomical Labeling 2 (31) and the Yeo-7 functional network atlas (32), a set of theory-driven seed regions was initially defined for whole-brain functional connectivity analysis. The detailed information of these seed regions, including their anatomical labels, MNI coordinates, and corresponding functional network assignments, is provided in Supplementary Table S1. Pearson correlation coefficients were calculated between the time series of each seed and that of every other voxel in the brain, and then transformed into Z values using Fisher’s r-to-z transformation to generate individual Z-functional connectivity maps (33).

Baseline FC differences between patients with MCI and healthy controls were then used to identify additional disease-related abnormal brain regions. The combined set of predefined and FC-informed regions of interest was subsequently entered into Granger causality analysis in the randomized MCI groups to characterize statistically inferred directed associations between brain regions. For Granger causality analysis, the representative time series of each seed region was extracted by averaging the BOLD time series across all voxels within a 6-mm-radius spherical ROI centered at the peak MNI coordinate of each cluster identified in the baseline FC analysis (Table 1) and the theory-driven seed regions (34, 35). The averaged time series were then entered into the rsHRF toolbox (36) (implemented in SPM12) for GCA estimation to characterize statistically inferred directed associations between each pair of seed regions (37). And the resulting values were converted using Fisher’s r-to-z transformation before statistical testing.

**Table 1:** Brain regions showing significant baseline functional connectivity abnormalities in patients with MCI relative to healthy controls.

Cluster	ROIs	Network	Voxel size	Peak intensity	Peak MNI coordinates		
					x	y	z
Cluster 1	Frontal_Inf_Tri_R	FPN VAN	437 483	4.86005 4.80957	48.00 −42.00	33.00 9.00	6.00 3.00
Cluster 2	Insula_L						
Cluster 3	Insula_R	VAN	355	4.2706	30.00	15.00	12.00
Cluster 4	Frontal_Sup_Medial_L	DMN	253	−4.83093	0.00	42.00	45.00
Cluster 5	Parietal_Inf_R	FPN	260	−4.9021	57.00	−48.00	39.00
Cluster 6	Frontal_Sup_Medial_R	DMN	377	−6.66456	3.00	39.00	42.00
Cluster 7	Calcarine_L	VIS	291	−4.38755	−12.00	−69.00	15.00
Cluster 8	Precuneus_L	DMN	183	−5.44237	−15.00	−54.00	54.00
Cluster 9	Cuneus_L	VIS	347	−4.44534	−18.00	−60.00	21.00
Cluster 10	Temporal_Mid_L	DMN	230	4.23300	−66.00	−18.00	−12.00
Cluster 11	Temporal_Pole_Mid_L	LIM	235	4.23572	−45.00	21.00	−27.00
Cluster 12	Temporal_Inf_R	DAN	250	−4.81391	45.00	9.00	−36.00
Cluster 13	Thalamus_R	LIM	311	4.34979	9.00	−27.00	9.00
Cluster 14	Cingulum_Mid_L	VAN	286	−4.45347	−12.00	−9.00	42.00
Cluster 15	Occipital_Inf_L	VIS	178	−5.00708	−36.00	−66.00	−6.00
Cluster 16	Paracentral_Lobule_L	SMN	233	−3.37228	−3.00	−27.00	63.00
Cluster 17	Occipital_Sup_L	VIS	209	−4.33792	−18.00	−87.00	27.00

The table lists brain regions showing significant baseline functional connectivity abnormalities in patients with MCI compared with healthy controls, together with their corresponding functional network assignments, voxel sizes, peak intensities, and MNI coordinates. These regions were subsequently used as seed regions for effective connectivity analysis in the randomized MCI groups. Patients with MCI exhibited significant functional connectivity alterations in the right pars triangularis of the inferior frontal gyrus (Frontal_Inf_Tri_R), left insula (Insula_L), right insula (Insula_R), left medial superior frontal gyrus (Frontal_Sup_Medial_L), right inferior parietal lobule (Parietal_Inf_R), right medial superior frontal gyrus (Frontal_Sup_Medial_R), left calcarine fissure (Calcarine_L), left precuneus (Precuneus_L), left cuneus (Cuneus_L), left middle temporal gyrus (Temporal_Mid_L), left middle temporal pole (Temporal_Pole_Mid_L), right inferior temporal gyrus (Temporal_Inf_R), right thalamus (Thalamus_R), left middle cingulate cortex (Cingulum_Mid_L), left inferior occipital gyrus (Occipital_Inf_L), left paracentral lobule (Paracentral_Lobule_L), and left superior occipital gyrus (Occipital_Sup_L). Frontoparietal network (FPN); ventral attention network (VAN); default mode network (DMN); visual network (VIS); limbic network (LIM); dorsal attention network (DAN); somatomotor network (SMN).

The imaging comparison between patients with MCI and healthy controls was observational and was performed only to identify baseline abnormalities relevant to MCI. The randomized treatment comparison was restricted to the taVNS and sham taVNS groups.

### 2.10 Statistical analysis

Statistical analyses were performed using SPSS Statistics 26. Demographic and clinical data from participants with complete baseline and follow-up assessments were included in the final analysis. Accordingly, the main efficacy analyses were conducted on a per-protocol basis.

#### 2.10.1 Clinical and demographic comparisons

For continuous variables meeting normality assumptions, independent-samples t tests were used for between-group comparisons and paired t tests for within-group comparisons, with results reported as mean ± standard deviation. For non-normally distributed continuous variables, the Mann–Whitney U test and Wilcoxon signed-rank test were used as appropriate. Categorical variables were compared using chi-square tests. All tests were two-tailed, and *p* < 0.05 was considered statistically significant (38). The primary efficacy analysis evaluated the between-group difference in change in MoCA-B score from baseline to week 12. Secondary efficacy analyses examined between-group differences in changes in the remaining neuropsychological and emotional scales. In addition to descriptive within-group pre-post comparisons, repeated-measures analysis of variance was used to assess group × time interaction effects, followed by *post-hoc* analyses when appropriate (39).

#### 2.10.2 Whole-brain functional connectivity and granger causality analyses

For both the baseline whole-brain FC comparisons between MCI patients and healthy controls, and the pre-to-post intervention GCA assessing changes in effective connectivity, multiple comparison correction was performed using Gaussian Random Field (GRF) theory at a voxel-level threshold of *p* < 0.001 and a cluster-level threshold of *p* < 0.05, two-tailed, to control the family-wise error rate (40, 41).

#### 2.10.3 Exploratory correlation analyses between GCA changes and clinical scores

For the exploratory correlation analyses between changes in Granger causality connectivity strength and changes in clinical scale scores, False Discovery Rate (FDR) correction was applied with q < 0.05. Results surviving FDR correction are reported as primary findings; uncorrected results (*p* < 0.05) are explicitly labeled as exploratory and interpreted with caution.

## 3 Results

### 3.1 Participant flow

**Figure 1:**
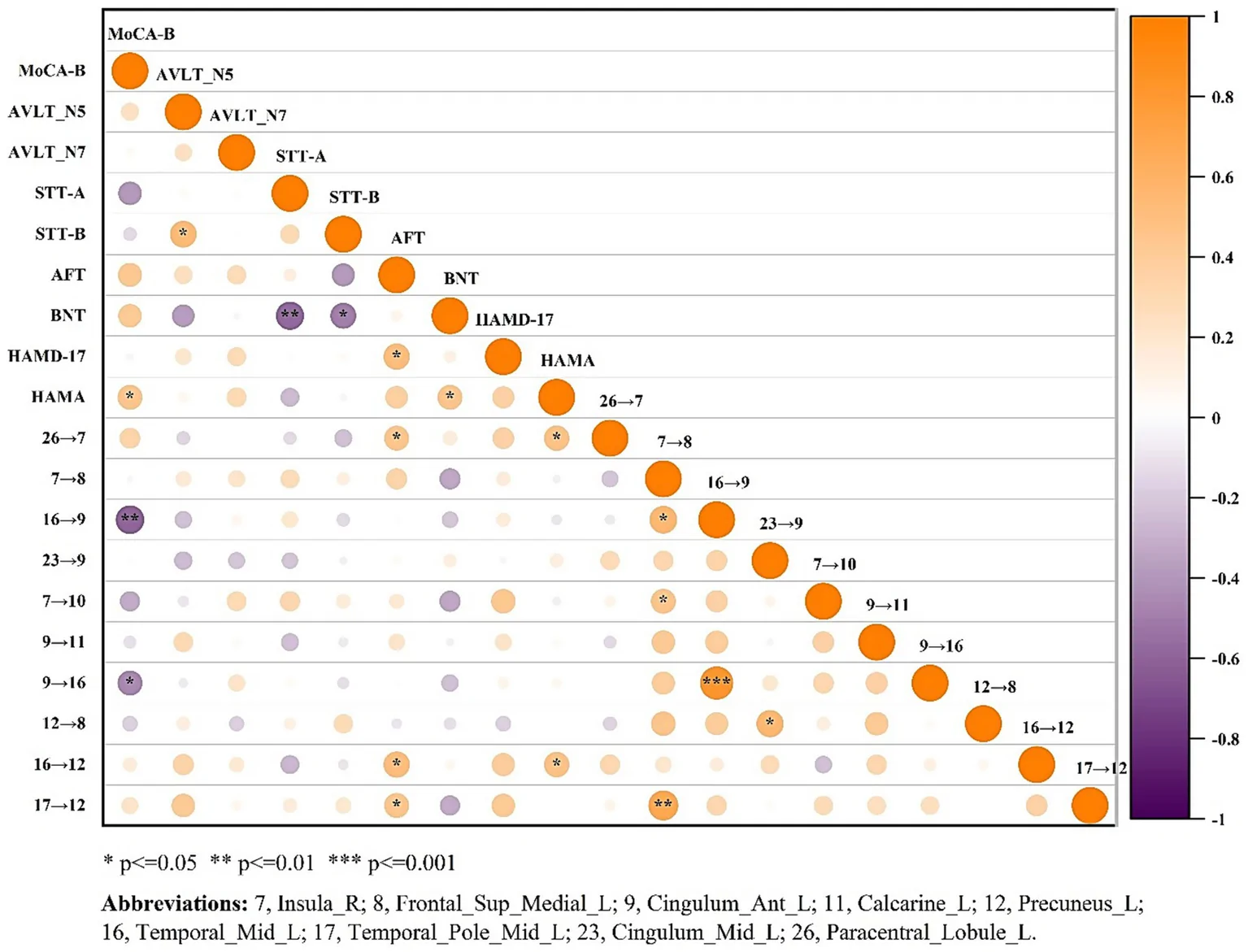
Matrix of scatter plots depicting the correlations between post-intervention changes in Granger causality (GC) connectivity strength (ΔGC) and changes in clinical scale scores (Δscores) in taVNS group. For each pair of brain regions, ΔGC strength (x-axis) is plotted against ΔMoCA-B, ΔAVLT_N5, ΔAVLT_N7, ΔSTT-A, ΔSTT-B, ΔAFT, ΔBNT, ΔHAMD-17, or ΔHAMA (y-axis). The color bar transitions from purple (negative) to orange (positive) to indicate Pearson correlation coefficients (*r* values), with significance levels denoted by * *p* < 0.05, ** *p* < 0.01, and *** *p* < 0.001. Significant correlations identified: Temporal. Mid. L → Cingulum. Ant. L with ΔMoCA-B (*r* = −0.591); Cingulum. Ant. L → Temporal. Mid. L with ΔMoCA-B (*r* = −0.448); Paracentral. Lobule. L → Insula. R with AFT (*r* = 0.454); Temporal. Mid. L → Precuneus. L with AFT (*r* = 0.500); Temporal. Pole. Mid. L → Precuneus. L with AFT (*r* = 0.454); Paracentral. Lobule. L → Insula. R with HAMA (*r* = 0.479); Temporal. Mid. L → Precuneus. L with HAMA (*r* = 0.464).

A total of 60 patients with MCI were assessed for eligibility, and all met the inclusion criteria. No participants were excluded before randomization. All 60 eligible patients were randomized in a 1:1 ratio, with 30 allocated to the taVNS group and 30 to the sham taVNS group. During follow-up, 1 participant in the taVNS group and 3 in the sham taVNS group were lost to follow-up. Among participants with follow-up data, no patient in the taVNS group was excluded from imaging analysis, whereas 4 patients in the sham taVNS group were excluded because of unusable imaging data. Ultimately, 29 patients in the taVNS group and 23 in the sham taVNS group were included in the final per-protocol analysis. Post-hoc sensitivity analysis based on these sample sizes revealed a minimum detectable effect size of Cohen’s d = 0.78 (two-tailed, *α* = 0.05, power = 0.80), which falls within the range of a large effect size, indicating sufficient statistical power to detect medium-to-large effects for the primary outcome. To further examine whether the treatment effect was influenced by baseline cognitive severity, we performed a post-hoc analysis of covariance (ANCOVA) with baseline MoCA-B score as a continuous covariate and the change in MoCA-B as the dependent variable. The results showed no significant group × baseline MoCA-B interaction (*p* > 0.05), indicating that the therapeutic effect of taVNS did not depend on baseline cognitive severity. For the primary outcome, the between-group difference in MoCA-B change from baseline to week 12 was 2.91 (95% CI: 0.79 to 5.03), with a Cohen’s d of 0.59, indicating a moderate-to-large effect size. To further assess the statistical power specifically for the within-group correlation analyses (Figure 1), we performed an additional *post-hoc* sensitivity analysis limited to the taVNS group (*n* = 29). Using G*Power for a two-tailed Pearson correlation test with α = 0.05 and power (1-*β*) = 0.80, the minimum detectable correlation coefficient (r) was calculated to be 0.367. All significant correlations identified in the exploratory analysis (r values ranging from −0.591 to 0.500, absolute values all > 0.367) exceeded this threshold, indicating that the current single-group sample size provided sufficient statistical power to detect the observed moderate-to-large effect sizes, which are hypothesis-generating in nature given the exploratory design. Baseline characteristics of completers versus non-completers/excluded participants are provided in Supplementary Table S2. To characterize the quality of the resting-state fMRI data, we computed head motion parameters for all included participants. The mean framewise displacement (FD) (42) and the mean and standard deviation of translational and rotational motion parameters are summarized in Supplementary Table S3. No significant differences were observed between the taVNS and sham taVNS groups in any of the motion parameters (all *p* > 0.05), indicating that group-level connectivity differences were unlikely to be confounded by differential head motion. The maximum translational motion across all included participants was below 2.0 mm, and the maximum rotational motion was below 2.0°, consistent with the pre-established exclusion criteria. In addition, 28 healthy controls were included as a non-randomized observational reference cohort for baseline imaging analyses.

### 3.2 Recruitment and baseline characteristics

Participants were recruited between January 2021 and December 2023. Follow-up assessments were completed after the prespecified 12-week intervention period, and the trial ended as planned. Baseline demographic and clinical characteristics of the randomized MCI groups are shown in Table 2. No significant differences were observed between the taVNS and sham taVNS groups in age, sex, education, disease duration, family history, or baseline neuropsychological and emotional scale scores (all *p* > 0.05), indicating good baseline comparability after randomization. The healthy control group was included only as a baseline imaging reference group and was not part of the randomized treatment comparison.

**Table 2:** Baseline demographic characteristics and pre- and post-treatment clinical scale scores in the randomized MCI groups, with healthy controls shown as a baseline reference group.

Characteristics	MCI				HCs	*P*_1_ value	*P*_2_ value	*P*_3_ value	*P*_4_ value
	taVNS (Pre)	taVNS(Post)	sham taVNS(Pre)	sham taVNS(Post)					
n	29	NA	23	NA	28	NA	NA	NA	NA
Age(y)	70.03 ± 6.48	NA	67.43 ± 5.10	NA	67.04 ± 5.11	0.056	0.076	NA	NA
Sex(M/F)	12/17	NA	7/16	NA	11/17	0.473	0.059	NA	NA
Education(y)	12.07 ± 2.85	NA	11.87 ± 2.99	NA	12.75 ± 3.30	0.404	0.152	NA	NA
Disease duration(m)	18.69 ± 9.73	NA	21.91 ± 17.10	NA	NA	0.213	NA	NA	NA
Family history	10/19	NA	5/18	NA	NA	0.314	NA	NA	NA
MoCA-B	19.31 ± 2.83	21.83 ± 2.28	19.39 ± 4.36	19.00 ± 5.07	NA	0.469	NA	<0.001	0.225
AVLT_N5	3.17 ± 2.28	4.07 ± 2.37	3.04 ± 1.92	3.74 ± 1.45	NA	0.413	NA	0.002	0.061
AVLT_N7	18.52 ± 4.69	19.90 ± 3.21	18.13 ± 6.68	18.65 ± 5.77	NA	0.408	NA	0.043	0.162
STT-A	114.93 ± 43.04	92.86 ± 28.34	125.17 ± 49.76	123.65 ± 50.06	NA	0.219	NA	<0.001	0.417
STT-B	288.45 ± 92.98	249.90 ± 85.74	308.22 ± 183.84	312.61 ± 180.64	NA	0.321	NA	<0.001	0.365
AFT	16.21 ± 4.62	17.97 ± 6.60	14.87 ± 4.76	14.13 ± 4.56	NA	0.157	NA	0.007	0.161
BNT	23.48 ± 3.61	25.24 ± 2.89	22.57 ± 4.18	21.87 ± 5.13	NA	0.204	NA	0.002	0.161
HAMD-17	1.52 ± 1.88	0.69 ± 0.89	1.61 ± 2.19	1.00 ± 0.90	NA	0.437	NA	0.006	0.069
HAMA	2.24 ± 1.75	1.34 ± 1.23	2.65 ± 1.77	2.13 ± 1.58	NA	0.204	NA	0.001	0.065

Data are presented as mean ± standard deviation (Mean ± SD). A *p*-value < 0.05 was considered statistically significant. p1 indicates the inter-group difference between the taVNS group and the sham taVNS group at baseline; p2 indicates the inter-group difference between patients and healthy controls at baseline; p3 indicates the intra-group difference before and after treatment in the taVNS group; p4 indicates the intra-group difference before and after treatment in the sham taVNS group. Healthy controls were included only as a baseline reference group for imaging analyses and were not part of the randomized treatment comparison. This table primarily presents baseline values and within-group pre-post comparisons. The prespecified primary outcome was the between-group difference in change in MoCA-B score, which should be interpreted in conjunction with the repeated-measures group×time analysis. The effect size (Cohen’s d) and 95% confidence interval for the primary outcome (MoCA-B change) are reported in Section 3.1 and Section 3.3. AFT, Animal Fluency Test; AVLT_N5, Auditory Verbal Learning Test-5th recall; AVLT_N7, Auditory Verbal Learning Test-7th recall/delayed recall; BNT, Boston Naming Test; HAMA, Hamilton Anxiety Rating Scale; HAMD-17, Hamilton Depression Rating Scale-17-item; sham taVNS, sham transcutaneous auricular vagus nerve stimulation (control group); taVNS, transcutaneous auricular vagus nerve stimulation; HCs, healthy controls; MCI, mild cognitive impairment; MoCA-B, Montreal Cognitive Assessment-Basic; STT-A, Shape Trails Test-Part A; STT-B, Shape Trails Test-Part B; y, years; m, months; M/F, male/female (sex distribution).

### 3.3 Clinical outcomes

After 12 weeks of intervention, the taVNS group showed significant within-group improvements in global cognition, memory, executive function, language function, and emotional status, as reflected by changes in MoCA-B, AVLT, STT, AFT, BNT, HAMD-17, and HAMA scores (all *p* < 0.05). In contrast, the sham taVNS group showed no significant within-group improvement on these clinical scales (all *p* > 0.05). Detailed numerical results are presented in Table 2.

The prespecified primary outcome was the between-group difference in change in MoCA-B score from baseline to week 12. Consistent with the overall pattern of clinical change, the taVNS group showed a more favorable cognitive response than the sham group, with a mean difference of 2.91 (95% CI: 0.79 to 5.03, Cohen‘s d = 0.59). Secondary clinical outcomes showed a similar pattern across multiple cognitive and affective domains. However, because Table 2 primarily presents baseline values and within-group pre-post comparisons, the between-group magnitude of change should be interpreted together with the repeated-measures group × time analysis described in the Statistical analysis section.

### 3.4 Baseline functional connectivity abnormalities in MCI

Baseline whole-brain functional connectivity analysis showed that, compared with healthy controls, patients with MCI exhibited significant FC abnormalities involving the right pars triangularis of the inferior frontal gyrus, bilateral insula, bilateral medial superior frontal gyri, right inferior parietal lobule, left calcarine fissure, left precuneus, left cuneus, left middle temporal gyrus, left temporal pole, right inferior temporal gyrus, right thalamus, left middle cingulate cortex, left inferior occipital gyrus, left paracentral lobule, and left superior occipital gyrus. These regions were mainly distributed across the default mode network, salience/attention-related systems, visual network, limbic-thalamic relay structures, and somatomotor-related regions (Table 1). These abnormal regions, together with predefined theory-driven regions of interest, were subsequently used for Granger causality analysis in the randomized MCI groups to examine intervention-related changes in effective connectivity.

### 3.5 Effective connectivity changes after intervention

**Figure 2:**
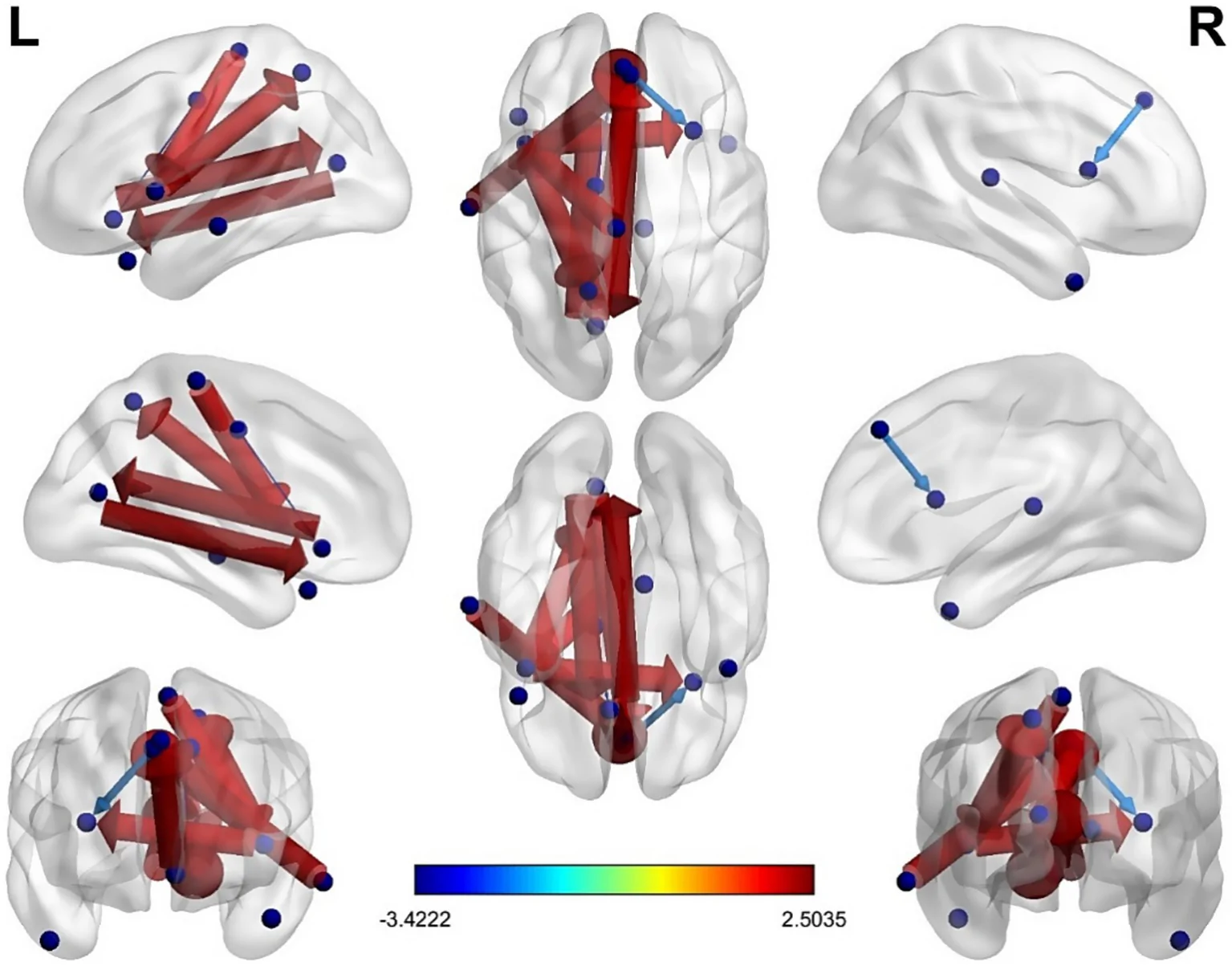
Alterations in effective connectivity in taVNS group after intervention based on Granger causality mapping (*p* < 0.05). Blue spherical nodes represent regions of interest (ROIs), and arrow lines indicate the direction of statistically inferred directed association from the source region to the target region. The color bar ranges from −3.4222 to 2.5035. Positive values (warm colors, e.g., red arrows) indicate increased connectivity, including Paracentral_Lobule_L → Insula_R, Insula_R → Frontal_Sup_Medial_L, Insula_R → Frontal_Sup_Medial_R, Temporal_Mid_L → Cingulum_Ant_L, Cingulum_Ant_L → Calcarine_L, Cingulum_Ant_L → Temporal_Mid_L, Temporal_Mid_L → Precuneus_L, and Temporal_Pole_Mid_L → Precuneus_L. Negative values (cool colors, e.g., blue arrows) indicate decreased connectivity, including Cingulum_Mid_L → Cingulum_Ant_L and Precuneus_L → Frontal_Sup_Medial_L.

**Figure 3:**
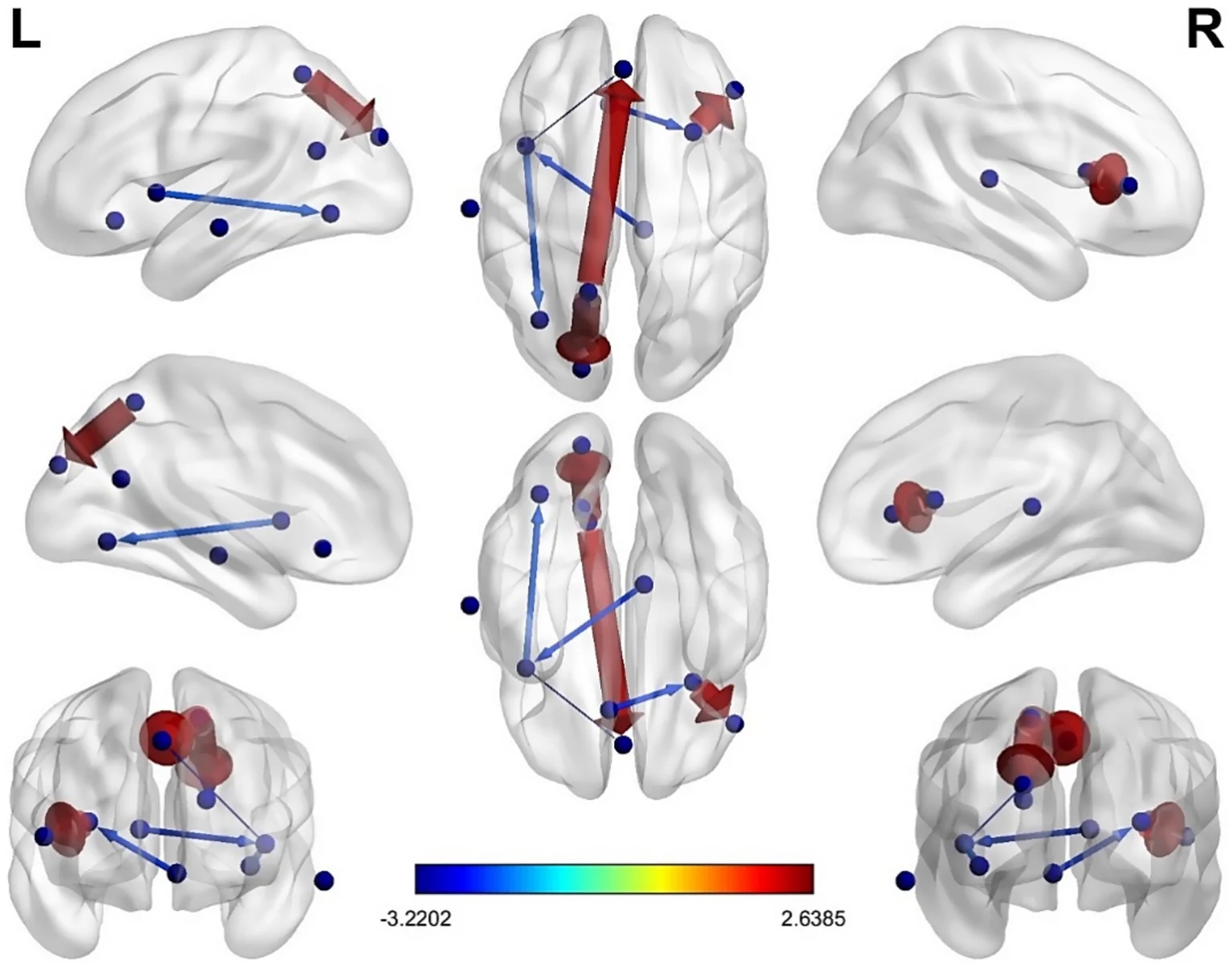
Alterations in effective connectivity in sham taVNS group after intervention based on Granger causality mapping (*p* < 0.05). Blue spherical nodes represent regions of interest (ROIs), and arrow lines indicate the direction of statistically inferred directed association from the source region to the target region. The color bar ranges from −3.2202 to 2.6385. Positive values (warm colors, e.g., red arrows) indicate increased connectivity, including Insula_R → Cuneus_L, Cingulum_Ant_L → Temporal_Mid_L, and Cingulum_Ant_L → Occipital_Sup_L. Negative values (cool colors, e.g., blue arrows) indicate decreased connectivity, including Frontal_Inf_Tri_R → Insula_L, Frontal_Sup_Medial_L → Insula_R, Insula_L → Temporal_Mid_L, and Insula_L → Occipital_Inf_L.

Granger causality analysis suggested distinct patterns of effective connectivity change after the intervention in the taVNS and sham taVNS groups. In the taVNS group, effective connectivity significantly increased in the following pathways: Paracentral_Lobule_L → Insula_R, Insula_R → Frontal_Sup_Medial_L, Insula_R → Frontal_Sup_Medial_R, Temporal_Mid_L → Cingulum_Ant_L, Cingulum_Ant_L → Calcarine_L, Cingulum_Ant_L → Temporal_Mid_L, Temporal_Mid_L → Precuneus_L, and Temporal_Pole_Mid_L → Precuneus_L (all *p* < 0.05). By contrast, effective connectivity significantly decreased in Cingulum_Mid_L → Cingulum_Ant_L and Precuneus_L → Frontal_Sup_Medial_L (all *p* < 0.05). In the sham taVNS group, effective connectivity significantly increased in Insula_R → Cuneus_L, Cingulum_Ant_L → Temporal_Mid_L, and Cingulum_Ant_L → Occipital_Sup_L (all *p* < 0.05), whereas significant decreases were observed in Frontal_Inf_Tri_R → Insula_L, Frontal_Sup_Medial_L → Insula_R, Insula_L → Temporal_Mid_L, and Insula_L → Occipital_Inf_L (all *p* < 0.05). Overall, compared with sham stimulation, taVNS was associated with a more organized and clinically relevant pattern of cross-network effective connectivity remodeling involving the insula, cingulate cortex, temporal cortex, and precuneus. Detailed results are shown in Figures 2, 3.

### 3.6 Correlation between imaging changes and clinical improvement

In the taVNS group, post-intervention changes in effective connectivity were significantly associated with changes in clinical scale scores (*p* < 0.05). Specifically, changes in MoCA-B score were negatively correlated with changes in Temporal_Mid_L → Cingulum_Ant_L connectivity (*r* = −0.591) and Cingulum_Ant_L → Temporal_Mid_L connectivity (*r* = −0.448). Changes in AFT score were positively correlated with changes in Paracentral_Lobule_L → Insula_R connectivity (*r* = 0.454), Temporal_Mid_L → Precuneus_L connectivity (*r* = 0.500), and Temporal_Pole_Mid_L → Precuneus_L connectivity (*r* = 0.454). Changes in HAMA score were positively correlated with changes in Paracentral_Lobule_L → Insula_R connectivity (*r* = 0.479) and Temporal_Mid_L → Precuneus_L connectivity (*r* = 0.464). These exploratory findings suggest that taVNS-related remodeling of effective connectivity may be associated with improvements in global cognition, semantic fluency, and anxiety symptoms in patients with MCI. Detailed correlation results are shown in Figure 1.

### 3.7 Adverse events

No serious adverse events were reported during the trial. No significant adverse reactions leading to treatment discontinuation were observed in either group.

## 4 Discussion

Using rs-fMRI, functional connectivity (FC), and Granger causality analysis (GCA), this study systematically examined the effects of transcutaneous auricular vagus nerve stimulation (taVNS) on clinical symptoms and large-scale brain network abnormalities in mild cognitive impairment (MCI). Four principal findings emerged. First, the taVNS group demonstrated a more favorable clinical response than the sham group; the prespecified primary outcome—the between-group difference in MoCA-B change from baseline to week 12—was consistent with a clinically meaningful effect of active stimulation (mean difference = 2.91, 95% CI: 0.79 to 5.03, Cohen’s d = 0.59). Significant within-group improvements across cognitive and affective scales were observed in the taVNS group but not in the sham group, providing supplementary support for the primary between-group comparison. Second, baseline FC revealed widespread MCI-related abnormalities involving the insula, medial frontal cortex, precuneus, temporal lobe, thalamus, cingulate cortex, and parieto-occipital regions, indicating distributed cross-network dysfunction. Third, pre-to-post intervention GCA showed that taVNS primarily reshaped directed information flow among the insula, anterior cingulate cortex, temporal cortex, and precuneus. In contrast, changes in the sham group were fewer, less coherent, and less clearly associated with clinical improvement. Fourth, in exploratory analyses applying False Discovery Rate (FDR) correction (q < 0.05), changes in several effective connectivity pathways were associated with changes in MoCA-B, AFT, and HAMA scores; these findings are hypothesis-generating and should be interpreted with caution given the exploratory nature of the analysis and the modest sample size. Together, these findings are consistent with the hypothesis that taVNS may be associated with changes in cross-network directed information flow centered on insula-, cingulate-, and precuneus-related pathways; however, they do not constitute definitive mechanistic evidence, and the observed patterns should be interpreted in the context of the study’s exploratory imaging analyses and methodological limitations.

### 4.1 Neuroimaging basis of baseline brain network abnormalities in MCI

Baseline FC results showed that patients with MCI exhibited significant functional abnormalities in the bilateral insula, medial superior frontal gyrus, precuneus, middle temporal gyrus, temporal pole, thalamus, middle cingulate cortex, and parts of the parieto-occipital cortex. These regions belong to, or are closely associated with, the default mode network (DMN) (43), salience network (SN), executive control-related networks (44), and the cortico-thalamic relay system (45). This pattern suggests that MCI is not confined to focal cortical impairment, but is more likely characterized by large-scale dysfunction arising from disrupted coordination across multiple networks (46). These findings are consistent with current neuroimaging evidence that MCI is an important transitional stage in the spectrum of Alzheimer’s disease and related neurodegenerative disorders, with impaired network-level integration as a core pathophysiological feature (47, 48).

Among these regions, the precuneus is a key DMN hub and is essential for self-referential processing, episodic memory retrieval, and internal information integration (49). Functional abnormalities of the precuneus have been repeatedly reported in MCI and early cognitive decline (43). The middle temporal gyrus and temporal pole are involved in semantic memory, emotional processing, and multimodal information integration, and dysfunction in these regions may contribute to impairments in language, memory, and emotion-related cognition (50). The insula and cingulate cortex are core SN nodes and are critically involved in salience detection, interoceptive monitoring, and switching between large-scale brain networks (51). The thalamus is a key subcortical relay structure linking sensory input with higher-order cortical processing (52). Together, these abnormal regions are unlikely to act in isolation and may instead form an interconnected pathological network underlying the clinical manifestations of MCI.

Although the hallmark features of MCI are deficits in memory and other cognitive domains, many patients also experience anxiety, depression, increased subjective cognitive burden, and disturbed internal state evaluation (53). Given the abnormalities observed in the insula, prefrontal cortex, cingulate cortex, and precuneus, the symptom profile of MCI may reflect not only disruption of memory-related networks, but also broader dysregulation involving self-referential processing, emotional regulation, salience evaluation, and cognitive control (8, 54). Accordingly, using the abnormal regions identified by baseline FC as seeds for subsequent GCA was both pathophysiologically meaningful and methodologically advantageous, allowing the analysis to move from spatially distributed abnormalities to altered directed information flow across networks.

### 4.2 Exploratory findings on taVNS-associated changes in cross-network directed information flow

From a neuroanatomical and neuromodulatory perspective, stimulation of the auricular afferent branch of the vagus nerve allows peripheral input to ascend through auricular afferents, the nucleus tractus solitarius (NTS), brainstem/thalamic relay nuclei, and ultimately limbic and cortical networks. Through this pathway, taVNS may influence the insula, anterior cingulate cortex, prefrontal cortex, thalamus, and limbic structures, all of which are closely involved in autonomic regulation, emotional processing, and higher-order cognition (55). Within this framework, the effects of taVNS in MCI are unlikely to be limited to local cortical excitability changes and may instead reflect systems-level modulation of large-scale network switching and information integration (56).

Within the taVNS group, pre-to-post intervention changes in effective connectivity were observed. These within-group changes should be considered supplementary to the primary between-group comparison, which provides the main evidence for treatment efficacy. Specifically, effective connectivity from Paracentral_Lobule_L to Insula_R increased, suggesting enhanced statistical directed association from sensorimotor-related input to the insula, a core hub of the SN. The insula plays a central role in integrating interoceptive signals, exteroceptive input, and salience evaluation, and is crucial for coordinating switching between the DMN and executive control-related networks (57). Strengthening of the Paracentral_Lobule_L → Insula_R pathway may suggest that peripheral stimulation-related signals were more effectively integrated into central salience-monitoring and sensory-integration systems, which is consistent with a potential basis for subsequent cross-network coordination, although this interpretation is based on within-group changes and should be considered hypothesis-generating (21). At the same time, effective connectivity from Insula_R to Frontal_Sup_Medial_L and Frontal_Sup_Medial_R was enhanced, suggesting enhanced directed association from the SN to medial prefrontal regions involved in higher-order control. Because the medial prefrontal cortex is implicated in self-referential processing, cognitive monitoring, emotional appraisal, and high-level resource allocation, these observed connectivity patterns are consistent with the possibility that taVNS may be associated with changes in the interaction between salience-monitoring and prefrontal regulatory systems; whether this reflects a direct mechanistic association on task organization or emotional introspection remains to be established (58).

Another within-group change observed after taVNS was the strengthening of the bidirectional pathways Temporal_Mid_L → Cingulum_Ant_L and Cingulum_Ant_L → Temporal_Mid_L. The anterior cingulate cortex is a key region for cognitive control, conflict monitoring, motivational processing, and emotional regulation (59), whereas the middle temporal gyrus contributes to semantic memory, episodic memory integration, and higher-order perceptual processing (60). These statistically inferred bidirectional changes in directed connectivity are consistent with the possibility that memory/semantic systems and cognitive-emotional integration systems may interact differently following taVNS, though whether this reflects a direct mechanistic effect or a correlate of broader network changes cannot be determined from the present data (61). This pattern of cognition-emotion co-regulation is clinically relevant, given the frequent coexistence of cognitive impairment and anxiety/depressive symptoms in MCI.

In addition, increased Temporal_Mid_L → Precuneus_L and Temporal_Pole_Mid_L → Precuneus_L connectivity suggests enhanced directed association from temporal regions involved in memory and semantic processing toward the precuneus, a central hub of the DMN (62). The precuneus is critically involved in episodic memory retrieval, self-referential processing, and internal cognitive integration, and its functional integrity is closely linked to global cognitive status (63). These observed within-group changes in directed connectivity, while secondary to the primary between-group comparison, are consistent with the hypothesis that temporal lobe–DMN pathways may be differentially engaged following taVNS, though direct evidence linking these statistical patterns to improved memory integration or self-related information processing at the neuronal level is lacking (64). Overall, these findings suggest that taVNS may be associated with changes in cross-network directed information flow centered on the insula/anterior cingulate cortex, precuneus, and related temporal pathways. These observed patterns of altered directed connectivity are consistent with the hypothesis that taVNS may be associated with changes in coordination among salience processing, cognitive control, and memory integration. However, these are exploratory findings, and the specific mechanisms underlying these associations require further investigation.

### 4.3 Exploratory findings suggest taVNS-associated changes may reflect reconfiguration of network connectivity patterns

Importantly, the within-group network changes observed following taVNS were not limited to increased connectivity. These within-group findings should be interpreted as supplementary to the primary between-group comparison. In addition to enhancement of several effective connectivity pathways, reductions in Precuneus_L → Frontal_Sup_Medial_L and Cingulum_Mid_L → Cingulum_Ant_L connectivity were also observed and may be mechanistically informative. These within-group findings suggest that the changes associated with taVNS may be interpreted as reflecting optimization or reconfiguration of pathological network organization, rather than nonspecific amplification of connectivity across the brain (65). In MCI, abnormal coupling between the DMN and prefrontal regions, inefficient compensatory recruitment, and pathological overengagement have all been reported (66). The reduced Precuneus_L → Frontal_Sup_Medial_L connectivity may therefore reflect suppression of abnormally elevated, inefficient, or compensatory directed information flow, allowing the network to shift toward a more balanced and efficient configuration (67). Similarly, reduced Cingulum_Mid_L → Cingulum_Ant_L connectivity may suggest correction of inefficient or maladaptive transmission patterns within the cingulate system (68).

In other words, the network reorganization observed following taVNS may involve two parallel processes: enhancement of pathways statistically associated with cognitive and emotional regulation (69), and suppression of inefficient, pathological, or unnecessary compensatory information flow, thereby improving overall network coordination (70). It should be emphasized that these GCA-derived patterns reflect statistical associations in directed connectivity rather than direct neuronal causal relationships. This distinction is important for understanding the mechanisms of neuromodulatory interventions. In MCI, network abnormalities often reflect not simply reduced regional activity, but disordered connectivity architecture (71) and suboptimal network configuration (72). Accordingly, therapeutically meaningful neuromodulation may not manifest as a global increase in brain activity, but rather as fine-tuned regulation of network topology and directed information flow (73). The coexistence of statistically increased and decreased directed connectivity pathways in the present study is consistent with the interpretation that taVNS may be associated with reconfiguration of network-level connectivity patterns. These exploratory findings are hypothesis-generating and do not constitute definitive evidence of systems-level network remodeling; whether such reconfiguration represents true restoration of dysfunctional organization requires validation in future studies with larger samples and multimodal designs.

### 4.4 Exploratory associations between connectivity changes and clinical improvements

The exploratory correlation analyses (FDR-corrected, q < 0.05) provided additional context for interpreting the clinical relevance of the neuroimaging findings. It should be noted that the primary evidence for clinical efficacy in this trial rests on the between-group difference in MoCA-B change as the prespecified primary outcome; the imaging–behavior correlations reported below were conducted within the taVNS group only and should be regarded as hypothesis-generating rather than confirmatory. In the taVNS group, changes in Temporal. Mid. L → Cingulum. Ant. L and Cingulum. Ant. L → Temporal. Mid. L connectivity were associated with improvements in MoCA-B scores after FDR correction, consistent with the possibility that restoration of bidirectional temporal–anterior cingulate coupling may be a potential imaging correlate of global cognitive improvement (74). Because the MoCA-B assesses multiple domains, including memory, executive function, attention, and language (75), this association may reflect coordinated multidomain improvement rather than changes in a single cognitive process (76). Changes in AFT scores were associated with increased connectivity in Paracentral. Lobule. L → Insula. R, Temporal. Mid. L → Precuneus. L, and Temporal. Pole. Mid. L → Precuneus. L, suggesting that improvements in verbal fluency and semantic retrieval may co-occur with coordinated changes in sensory integration, salience processing, and temporal lobe–DMN pathways (77). As the AFT is sensitive to semantic retrieval, executive control, and processing speed, its improvement may reflect broader coordination across multiple cognitive systems (78). In addition, changes in HAMA scores were associated with Paracentral. Lobule. L → Insula. R and Temporal. Mid. L → Precuneus. L connectivity, consistent with the possibility that the anxiolytic effects of taVNS involve reorganization of insula-mediated interoceptive processing and precuneus-related self-referential and emotional integration (79). Given the frequent coexistence of cognitive impairment and anxiety symptoms in MCI, these parallel improvements may reflect shared cross-network changes, though the directionality of these relationships cannot be established from the present data (11, 15).

These exploratory correlations indicate a statistical co-occurrence between brain network changes and clinical improvement that survived FDR correction; however, given the exploratory nature of these analyses, the relatively small sample size, and the inherent limitations of within-group post-hoc correlation testing, these findings should be interpreted with caution and not treated as confirmatory evidence. Replication in larger, prospectively powered independent samples is needed before any firm conclusions can be drawn (80). It is important to note that the GCA findings reflect statistical inference of temporal precedence in BOLD signal fluctuations rather than direct evidence of neuronal causality. Therefore, the GCA-derived patterns should be understood as statistically inferred directed associations between regional BOLD time courses—reflecting temporal precedence in signal fluctuations—rather than as evidence of synaptic or neuronal causal transmission. The observed directed connectivity patterns should therefore be interpreted as markers of coordinated network dynamics that accompany clinical improvement, rather than as definitive proof of causal influence between specific brain regions (81). Nevertheless, from the perspective of converging evidence linking peripheral neural stimulation, central network reorganization, and behavioral improvement (82), the present study provides a preliminary multilevel framework for understanding the effects of taVNS in MCI. This gives the findings greater explanatory value than studies reporting only clinical efficacy or only imaging differences.

### 4.5 Value of the combined FC and GCA approach

Methodologically, the combined use of FC and GCA is also a strength of this study. FC mainly reflects synchrony and correlation between time series of different brain regions and is useful for identifying abnormal regions and network distributions in disease states (46). GCA, by contrast, can partly characterize directed statistical associations between brain regions based on temporal precedence, thereby complementing FC in explaining how abnormal covariance patterns may shift toward reorganization of information transfer after intervention (23). Combining FC and GCA therefore extends the analysis from identifying where abnormalities occur to understanding how they are transmitted and reorganized after treatment. This framework is particularly relevant for MCI, in which network dysregulation is a core pathological feature [70]. Because the clinical manifestations of MCI are driven by impaired coordination across multiple functional systems rather than damage to a single region (83), FC alone can reveal synchronous alterations but cannot readily identify potential driving nodes or the direction of information flow. Applying GCA to regions identified as abnormal at baseline makes it possible to determine which pathways show statistically inferred changes after intervention and whether these changes are statistically associated with clinical improvements (84). Thus, the present strategy—first identifying abnormal regions and then characterizing directed patterns—balances disease relevance with mechanistic interpretability.

However, it is critical to acknowledge that GCA in fMRI studies reflects statistical inference of temporal precedence and predictive relationships in BOLD time series rather than direct neuronal causality (81). The term “effective connectivity” as measured by GCA should be understood as a data-driven characterization of statistical associations between regional time courses, not as definitive evidence of synaptic or axonal connectivity. Its results may be influenced by multiple confounding factors, including sampling rate, hemodynamic variability, preprocessing strategy, and region definition (81). Therefore, the GCA findings in this study should be interpreted cautiously as changes in statistically inferred directed information flow between brain regions, which may reflect coordinated network dynamics accompanying taVNS intervention, rather than as strict biological proof of causal neural transmission (85). Although GRF and FDR corrections were applied to control type I errors, they do not completely eliminate false-positive findings, particularly given the large number of connectivity comparisons in this exploratory study. GRF controls the cluster-level family-wise error rate, whereas FDR controls the false discovery rate; however, both rely on statistical assumptions and may not fully account for the complex spatial and temporal dependencies in rs-fMRI data. Therefore, the significant findings—especially those from exploratory analyses—should be considered hypothesis-generating and require independent replication. More comprehensive validation of the mechanisms of taVNS in MCI will require future studies incorporating structural connectivity (e.g., diffusion tensor imaging), electrophysiological techniques with higher temporal resolution (e.g., EEG/MEG), direct measures of neural responsiveness to stimulation, and longitudinal multi-time-point designs (85).

The 6-min acquisition time, while consistent with prior studies, may have affected the precision of individual-level connectivity estimates. Longer acquisitions (≥10 min) are generally recommended for individual-level analyses to improve test–retest reliability (86, 87). Several factors mitigate this concern in the present study. First, our primary imaging inferences are based on group-level comparisons, which are less sensitive to within-subject variability. Second, all significant FC and GCA findings were identified using GRF cluster-level correction (*p* < 0.05), reducing the likelihood of noise-driven results. Third, the observed connectivity changes were systematically associated with clinical improvements, providing external validity. Nonetheless, the relatively short acquisition time remains a consideration, and future studies with longer scan durations are warranted.

### 4.6 Interpretation of findings in the sham taVNS group and the specificity of taVNS effects

The sham taVNS group did not show a complete absence of imaging changes, but rather several scattered and inconsistent connectivity alterations. This suggests that even without direct stimulation of the auricular vagal afferent pathway, nonspecific auricular stimulation, repeated measurements, expectancy effects, environmental adaptation, or natural disease fluctuation may still influence brain function to some extent (86). This phenomenon is not uncommon in neuromodulation studies and indicates that findings in the control group should not simply be dismissed as nonspecific effects (88).

However, compared with the taVNS group, the connectivity changes in the sham taVNS group did not form a coherent network pattern centered on the insula, anterior cingulate cortex, precuneus, and related temporal pathways, nor were they systematically coupled with clinical benefits. These findings support the interpretation that general auricular stimulation may induce limited short-term neural responses, whereas network-level reorganization associated with cognitive and emotional improvement more likely depends on effective activation of the vagal afferent pathway and its specific central regulatory effects (89). In other words, the specificity of taVNS may lie in its greater ability to induce brain network remodeling characterized by functional organization, cross-network consistency, and clinical relevance (90).

### 4.7 MCI heterogeneity and implications for treatment response

MCI is a heterogeneous clinical syndrome encompassing multiple etiological and clinical subtypes, including amnestic and non-amnestic variants, which may differ in their underlying neuropathology, cognitive trajectories, and responsiveness to interventions (91). In the present study, we did not perform stratified analyses by MCI subtype, as such analyses were not pre-specified and the current sample size precluded meaningful formal subgroup comparisons. However, to explore whether baseline cognitive severity influenced treatment response, we conducted a post-hoc ANCOVA with baseline MoCA-B score as a continuous covariate, which revealed no significant group × baseline MoCA-B interaction (*p* > 0.05). This suggests that the therapeutic effect of taVNS was not strongly dependent on baseline global cognitive severity within the range of our sample. Nevertheless, this does not exclude the possibility that treatment effects may differ across MCI subtypes with distinct neurobiological profiles (e.g., amnestic vs. non-amnestic, or biomarker-defined subgroups) (92, 93). The lack of biomarker data (e.g., amyloid-*β*, tau, APOE genotype) in the present study further limits our ability to characterize the underlying pathology of MCI in our sample (94). Future studies with larger samples and biomarker-stratified designs are warranted to determine whether taVNS exerts differential effects across MCI subtypes and to identify predictors of treatment response (99), given the exploratory nature of the present findings.

### 4.8 Limitations

Several limitations should be acknowledged. First, the final sample size was modest (95), which may have limited statistical power for detecting more subtle clinical and imaging effects and reduced the ability to examine heterogeneity within MCI. We acknowledge that the absence of a formal *a priori* sample size calculation remains a methodological limitation of this exploratory study. Although the post-hoc sensitivity analyses indicated sufficient power to detect the observed medium-to-large effects, a pre-specified power calculation would have provided stronger justification for the sample size. Future studies with larger, multicenter samples and prospective sample size estimations are warranted to confirm these preliminary findings and to enable adequate subgroup analyses across different MCI subtypes. Second, to evaluate the potential impact of attrition, we compared baseline characteristics between completers (*n* = 52) and non-completers/excluded participants (*n* = 8). No significant differences were found in age, sex, education, or baseline MoCA-B (all *p* > 0.05), suggesting attrition was unrelated to baseline disease severity. A LOCF sensitivity analysis for the primary outcome also confirmed the robustness of the between-group difference (*p* < 0.05). These results are presented in Supplementary Table S2 and summarized in Section 3.1, with a citation in Section 5. Third, rs-fMRI-based Granger causality analysis reflects directed statistical dependence in BOLD time series rather than direct neuronal causality, and the findings may be influenced by hemodynamic delay, temporal resolution, preprocessing strategy, and region definition (87). Therefore, the GCA-derived results in this study should be interpreted as statistically inferred directed associations that may reflect coordinated network dynamics accompanying taVNS intervention, rather than as definitive evidence of causal neural transmission. In addition, despite the use of GRF and FDR corrections, the large number of connectivity comparisons precludes the complete exclusion of false-positive findings. Thus, the imaging results should be interpreted as exploratory and hypothesis-generating, rather than confirmatory. Fourth, only short-term post-treatment effects were assessed, and the durability of clinical and imaging changes remains unknown (96). Fifth, MCI is a heterogeneous syndrome, and the present sample size did not allow stratified analyses by MCI subtype, biomarker status, or baseline symptom profile (97). Although a post-hoc ANCOVA suggested that the treatment effect did not significantly depend on baseline MoCA-B scores, this does not exclude the possibility of differential effects across MCI subtypes with distinct neurobiological characteristics. Future studies with larger, multicenter samples are warranted to perform adequately powered subgroup analyses stratified by MCI subtype and biomarker profiles, which will help to identify patients who are most likely to benefit from taVNS and to elucidate the specific network mechanisms underlying treatment response across different MCI subtypes [102]. Sixth, the resting-state fMRI acquisition time was 6 min, which is relatively short compared to the 10–15 min recommended for individual-level connectivity analyses [100]. While group-level analyses are less affected by this limitation and our key findings survived rigorous multiple-comparison correction, the shorter scan duration may have reduced the reliability of individual-level functional and effective connectivity estimates. Future studies with longer acquisition times are needed to confirm the stability of these connectivity measures at the individual level. Future studies with longer acquisition times are needed to confirm the stability of these connectivity measures at the individual level. Future studies with larger, multicenter samples are warranted to perform adequately powered subgroup analyses stratified by MCI subtype and biomarker profiles, which will help to identify patients who are most likely to benefit from taVNS and to elucidate the specific network mechanisms underlying treatment response across different MCI subtypes (98).

## 5 Conclusion

This study provides preliminary, hypothesis-generating evidence consistent with the possibility that taVNS may be associated with improvements in cognition and emotion in MCI, and that these improvements are accompanied by changes in cross-network transfer centered on the insula, anterior cingulate, precuneus, and temporal regions. While these observed associations are consistent with a potential role for coordinated changes in salience, memory, self-referential processing, and emotion regulation, the current findings do not establish definitive causal mechanisms and require confirmation in future studies. These findings offer neuroimaging support for taVNS in MCI and a basis for future individualized neuromodulation guided by brain network biomarkers.

## Data Availability

The original contributions presented in the study are included in the article/Supplementary material, further inquiries can be directed to the corresponding authors.

## Footnotes

1
https://www.fil.ion.ucl.ac.uk/spm/

2
http://rfmri.org/dpabi
