# Supplementary material for: Rs-fMRI reveals that taVNS improves cognitive and emotional function in mild cognitive impairment by reconfiguration of “sensory relay-salience-default” cross-network effective connectivity: a randomized, double-blind, sham-controlled trial
Source: Front Neurol. 2026 Aug 12;17:1891440. doi: 10.3389/fneur.2026.1891440 (PMC13506392; doi:10.3389/fneur.2026.1891440)
Supplement: Supplementary file 1 [file Table_2.docx]

Supplementary Table S2. Comparison of baseline characteristics between completers and non‑completers/excluded participants, and sensitivity analysis of the primary outcome under last‑observation‑carried‑forward (LOCF) imputation

| Variables | Completers (n = 52) | Excluded (n = 8) | *P* value |
| --- | --- | --- | --- |
| Age (years) | 68.92 ± 6.03 | 68.25 ± 5.89 | 0.763 |
| Sex (Male/Female) | 19/33 | 3/5 | 0.892 |
| Education (years) | 11.96 ± 2.87 | 12.13 ± 3.02 | 0.875 |
| Baseline MoCA‑B | 19.35 ± 3.48 | 19.25 ± 4.01 | 0.938 |
| LOCF |  |  |  |
| MoCA‑B change from baseline | taVNS: +2.52 ± 2.15  Sham: −0.39 ± 3.12 |  | < 0.001 |

Note:Data are presented as mean ± standard deviation (Mean ± SD) or as counts (n). No significant differences were observed between completers and non‑completers/excluded participants for any baseline variable (all *P* > 0.05), suggesting that attrition was unlikely to be systematically related to baseline disease severity or key prognostic factors. Under LOCF imputation for the primary outcome, the between‑group difference in MoCA‑B change remained statistically significant (*P* < 0.05), supporting the robustness of the primary conclusion to missing data assumptions.
