# Supplementary material for: Rs-fMRI reveals that taVNS improves cognitive and emotional function in mild cognitive impairment by reconfiguration of “sensory relay-salience-default” cross-network effective connectivity: a randomized, double-blind, sham-controlled trial
Source: Front Neurol. 2026 Aug 12;17:1891440. doi: 10.3389/fneur.2026.1891440 (PMC13506392; doi:10.3389/fneur.2026.1891440)
Supplement: Supplementary file 2 [file Table_3.docx]

**Supplementary Table 3. Head motion parameters for the taVNS and sham taVNS groups.**

| Motion parameter | taVNS group (n = 29) | **Sham taVNS group (n = 23)** | *P* value |
| --- | --- | --- | --- |
| Mean FD_Power (mm) | 0.205 ± 0.099 | 0.223 ± 0.124 | 0.623 |
| Max translation (mm) | 0.597 ± 0.337 | 0.614 ± 0.429 | 0.893 |
| Max rotation (°) | 0.527 ± 0.170 | 0.590 ± 0.361 | 0.498 |
| Mean translation (mm) | 0.241 ± 0.180 | 0.268 ± 0.229 | 0.693 |
| Mean rotation (°) | 0.163 ± 0.085 | 0.201 ± 0.131 | 0.301 |

Note:Data are presented as mean ± standard deviation. FD, framewise displacement. For each participant, mean FD and motion parameters were averaged across pre‑ and post‑intervention scans (or the available scan if the other time point was excluded). Participants exceeding 2.0 mm in translation or 2.0° in rotation at either time point were excluded from the final analysis. No significant differences were observed between the two groups for any motion parameter (independent‑samples t‑tests, all P > 0.05), indicating that group‑level connectivity differences were unlikely to be confounded by differential head motion.
